# The Impact of Smart Materials in Restorative Dentistry and Endodontics From Innovation to Application: A Narrative Review

**DOI:** 10.7759/cureus.82858

**Published:** 2025-04-23

**Authors:** Mausmee Ved, Niraj Kinariwala, Abhishek Singh, Dhwani Bhatia, Huma Shaikh, Zalak Padmani, Tulsi Raja, Nishtha Panchal

**Affiliations:** 1 Conservative Dentistry and Endodontics, Karnavati School of Dentistry, Gandhinagar, IND; 2 Dentistry, Karnavati School of Dentistry, Gandhinagar, IND

**Keywords:** self healing composites, smart bur, smart composites, smart dentistry, smart glass ionomer cement, smart material

## Abstract

Smart materials have transformed restorative dentistry and endodontics by developing materials that adapt dynamically to external stimuli, including pH, temperature, and mechanical stress. The term "smart" materials refers to the fact that certain materials can be designed to react to many stimuli, including temperature, humidity, pH, stress, electric fields, and magnetic fields. Their increased biocompatibility, prolonged stress tolerance, sealability, and antibacterial qualities make them suitable for use as cement, root restoration materials, root canal sealing agents, filling materials, and sustainable, aesthetically pleasing, and reconstructive products. Making intelligent materials for dentistry is an idea that, such as dentine or enamel, has drawn interest.

These materials could lead to innovative and revolutionary dental procedures with significantly better clinical results. An assortment of medications and irrigants are utilized to eradicate canal microbes. To solve this problem, antibacterial nanoparticles have been created. In order to improve general oral health, nanomaterials and dental nanorobots are used in diagnostic and therapeutic procedures. The purpose of this study is to demonstrate how "smart materials" can be used in dentistry to maximize the benefits of traditional restorative procedures.

## Introduction and background

Dental materials must, above all, be compatible with the fluids present in the oral cavity, such as gingival crevicular fluid and saliva. Among bioactive materials are the most reliable and durable [1]. Smart materials are being created in search of ideal materials, which may result in smart dentistry. There was never a dental material that was perfect and satisfied every need for an exceptional material [1]. They are known as intelligent materials because, in accordance with Takagi (1990), they react to changes in the environment under optimal conditions and consequently disclose their own roles [1]. Dental materials were designed to be inactive and neutral, which means they have little contact with body tissues and substances [1]. Several smart materials have been introduced in dentistry, including composites, smart ceramics, amorphous calcium phosphate that releases pit and fissure sealants, orthodontic shape memory alloy wire, smart sutures, and smart burs. Three functions can be distinguished in material intelligence: processing of sensed information, detecting changes in the environment, and ultimately making decisions by responding to the stimuli [2]. Magnetostrictive technology, during World War I, assisted the Coalition in locating German U-boats by using nickel as an acoustic source and was the first application of smart materials [2]. According to some academics, no material is genuinely intelligent on its own; it is just receptive [2]. A number of materials are already available based on their strength and biocompatibility [2]. Modern dental tools have been enhanced to become more sophisticated and knowledgeable. In recent years, dentistry has improved because of these innovative materials [2].

## Review

Methodology

This review used Internet resources like PubMed, Google Scholar, Web of Science, and EBSCOhost to meticulously collect literature on the use of smart materials. The search terms that were used were "smart materials," "smart dentistry," "smart glass ionomer cement," "self-healing composites," and "smart composites." Relevant books, articles, and reviews were among the requirements for inclusion. Titles and abstracts were screened as part of the study selection process, and then relevant papers were evaluated in full. The last set of included studies provides a thorough analysis of the existing data regarding the employment of nanotechnology in dentistry.

Smart material classification

Smart materials can be categorized into the following groups: a) passive materials, including dental composites and glass ionomers modified with resin, and b) active materials like smart ceramics and smart composites.

Passive materials respond to environmental changes when they are not under external control. Also, they are capable of self-healing [3]. Characteristics are when mechanical stress is applied, piezoelectric devices generate an electric current; shape memory: these materials can remember their original shape after deformation and regain it when heated; conversely, thermochromic materials change color in reaction to temperature variations; materials that change color in response to changes in illumination are known as photochromic materials; when exposed to a magnetic field, fluids known as magnetorheological materials harden; and materials are considered pH-sensitive if they swell or collapse in response to changes in the surrounding media's pH biofilm formation - the interaction between a material's surface and its environment is altered when biofilm is present [3].

Mechanism of Smart Materials

These compounds have instructive and formative effects on both tissues and cells to support tissue regeneration and repair by responding to both internal and external factors, such as temperature, ionic strength, magnetic field, and pH. In order to actively participate in tissue regeneration, it has cleverly and carefully modified human functions [3,4].

Criteria for a Smart Material

Nature of inequality, absorption in or reacting to impulses, add a minimum of one item with an appropriate structure [4].

By Structure

These methods are to be applied to materials containing a polysalt matrix to integrate and produce intelligent behavior [4].

Water's role: Intelligent behavior is associated with a structure's ability to rapidly release or absorb solvent in response to temperature stimulation. Depending on the characteristics of the water and the rigidity of the bonds that are present, the structure's dimensional stability may be reduced or increased [4]. The behavior of heat: The way a compound behaves thermally is mostly determined by its coefficient of thermal expansion. They expand and contract more than teeth that are naturally occurring [4]. Radial pressure and expansion: Resins can be added to the salts and gel structure to stabilize the component's strength and lifespan [4].

Amorphous calcium phosphate (ACP)

ACP serves as a precursor in the biological synthesis of hydroxyapatite (HAP). Because of its restorative and preventative qualities, it is used in adhesives, dental cement, composites, and pit and fissure sealants [5,6]. ACP and a milk component called casein phosphopeptide (CPP) combine to create a material that dentifrices employ as a remineralizing agent [6].

Fluoride-releasing pit and fissure sealant

Pit-and-fissure sealants are a useful component of a thorough caries prevention strategy [7]. Recent studies have demonstrated that placing sealants on early, non-cavitated carious lesions can also be an effective secondary preventative approach, even though they have typically been employed to prevent primary caries [7]. Glass ionomer cement and resin-based sealants are the two main categories of readily available pit-and-fissure sealing products. The two processes, auto polymerization, and visible light photopolymerization, can be combined to polymerize readily available resin-based sealing materials [8].

Smart composites

With an aluminum composite panel filler encased in a polymer binder, SKRTIC (Dun & Bradstreet, Jacksonville, Florida, USA) developed innovative biologically active restorative materials that may promote tooth structure regeneration by gradually releasing significant amounts of calcium and phosphate ions [9]. When intraoral pH levels fall below the essential pH of 5.5, this light-activated alkaline, nano-filled glass restorative material releases calcium, fluoride, and hydroxyl ions to help remineralize teeth and prevent tooth surface demineralization [9]. It is possible to sufficiently cure up to 4 mm of the material's bulk thickness. For both primary and permanent teeth, it is advised for the recovery of class I and class II lesions [10].

**Figure 1:**
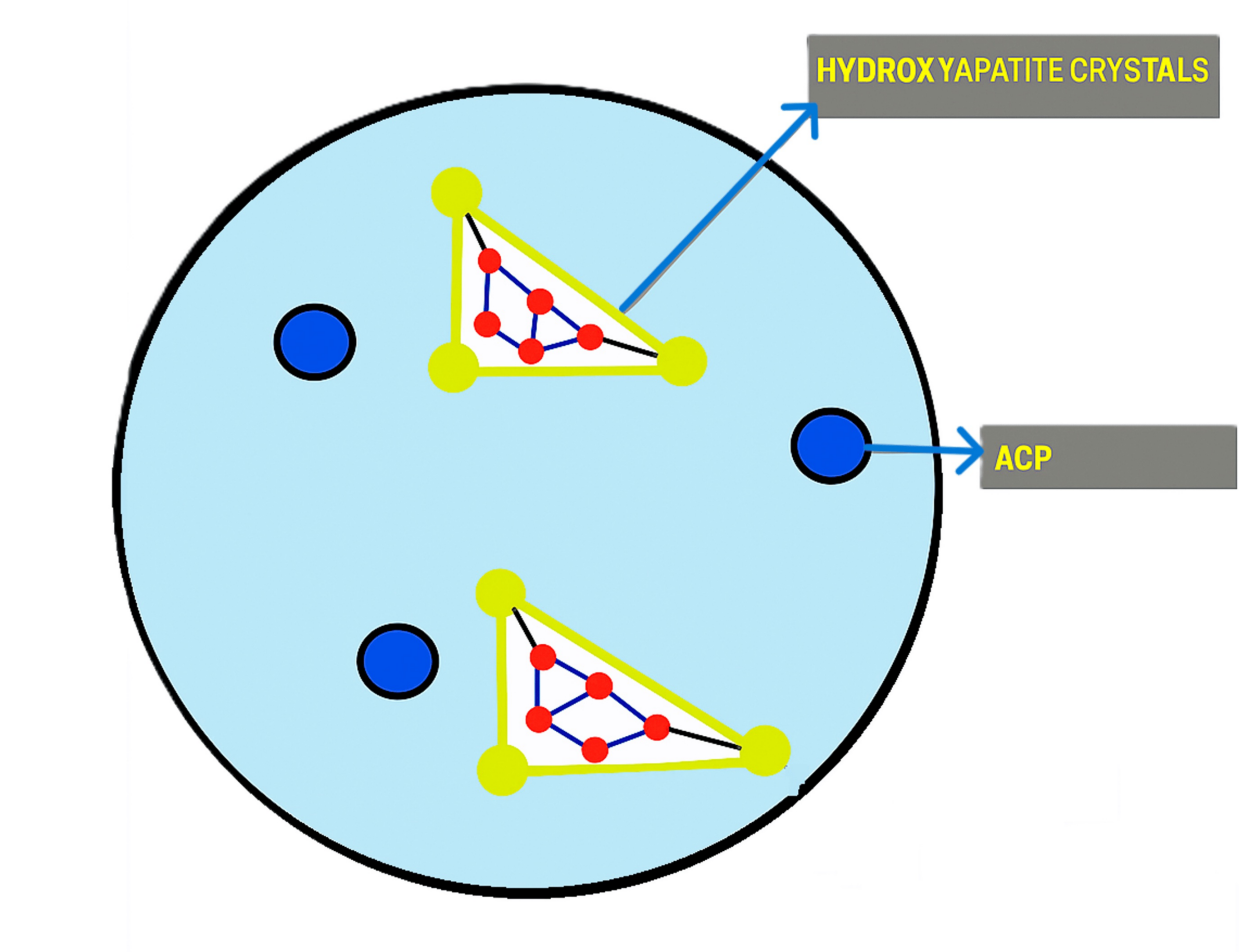
Formation of hydroxyapatite crystals. The image is created by, Mausmee Ved, the author of this article using Microsoft Paint. Microsoft Paint (Redmond, USA)

Amorphous calcium phosphate (ACP), one of the most soluble of the physiologically important calcium phosphates, is present in smart composites and transforms into crystalline hydroxyapatite (HAP) the quickest [10]. ACP will function as a long-term releasing agent when combined with meticulously designed and produced resins to form a composite material [11]. Calcium and phosphate supply will aid in preventing dental cavities. ACP has been investigated as a filler phase in bioactive polymeric composites [12] (Figure 1).

Self-cured composites

**Figure 2:**
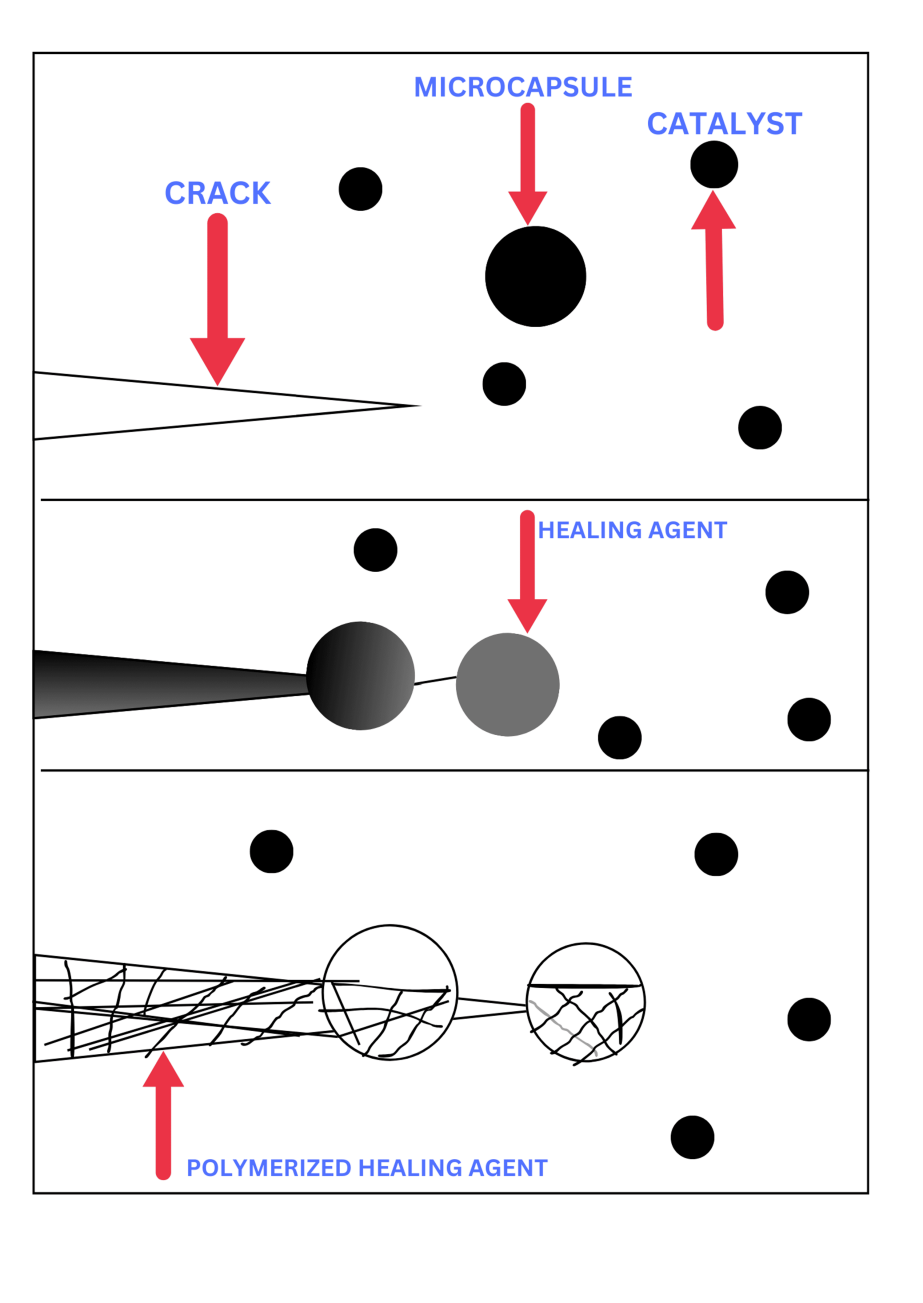
The self-healing composite's microcapsule approach mechanism. The image is created by, Mausmee Ved, the author of this article using Microsoft Paint Microsoft Paint (Redmond, USA)

It's interesting to note that one of the earliest synthetic materials to be claimed to be self-repairing or self-healing shares some characteristics with resin-based dental materials [13]. If the epoxy composite structure breaks, some of the microcapsules that are loaded with resin in this epoxy system will disintegrate near the point of breakage and expose the resin [14]. After filling the crack, the resin interacts with a Grubbs catalyst that has been added to the epoxy composite, polymerizing the resin and sealing the crack [15] (Figure 2).

Application of smart dental bonding system

Dental adhesives are now being given intelligence by adding silver nanoparticles, ACP, and DMADDM (dimethylamino dodecyl methacrylate), which give them antimicrobial and self-repairing properties [16]. Research has shown that ACP eliminates calcium and phosphate ions, which may aid in the remineralization of dental lesions. Silver nanoparticles and DMADDM drastically lower the biofilm and its metabolic processes, preventing subsequent caries [17].

Smart glass ionomer cement (RMGIs)

Following extensive research on the coefficient of thermal expansion, it was discovered that glass ionomer cement (GIC) may exhibit intelligent, thermoresponsive behavior [18]. Davidson initially proposed the clever actions of GIC. Consequently, it can be said that the glass ionomer materials are intelligent enough to mimic the characteristics of human dentine [18]. The fluoride release and recharge capacity of these materials is the other component of their intelligent behavior. These clever properties are also observed in glass ionomer cement, and compomer [19]. For example, GC Fuji IX EXTRA (© GC, America Inc.).

Smart ceramic

These biocompatible, metal-free, and realistic restorations look much like real teeth. For example, the Cercon Zirconium Smart Ceramic System (Dentsply Sirona, Charlotte, NC) made it simple and predictable to return teeth to their original shape [20]. At ETH Zurich, the first all-ceramic tooth bridge was created in 1995 using a technique that allowed ceramic teeth and bridges to be machined directly [20]. The process and materials have since undergone testing and have been approved as Cercon. Because of Cercon's strength and experience, the bridge may be constructed without the use of metal or stainless steel. The end result is a realistic-looking, biologically compatible, metal-free repair with strength that helps keep cracks from developing [21]. Artificial grey shadows from the underlying metal and ugly dark edges are no longer an issue with Cercon. It is widely utilized in implants and other non-metal applications [22].

Smart prep burs

**Figure 3:**
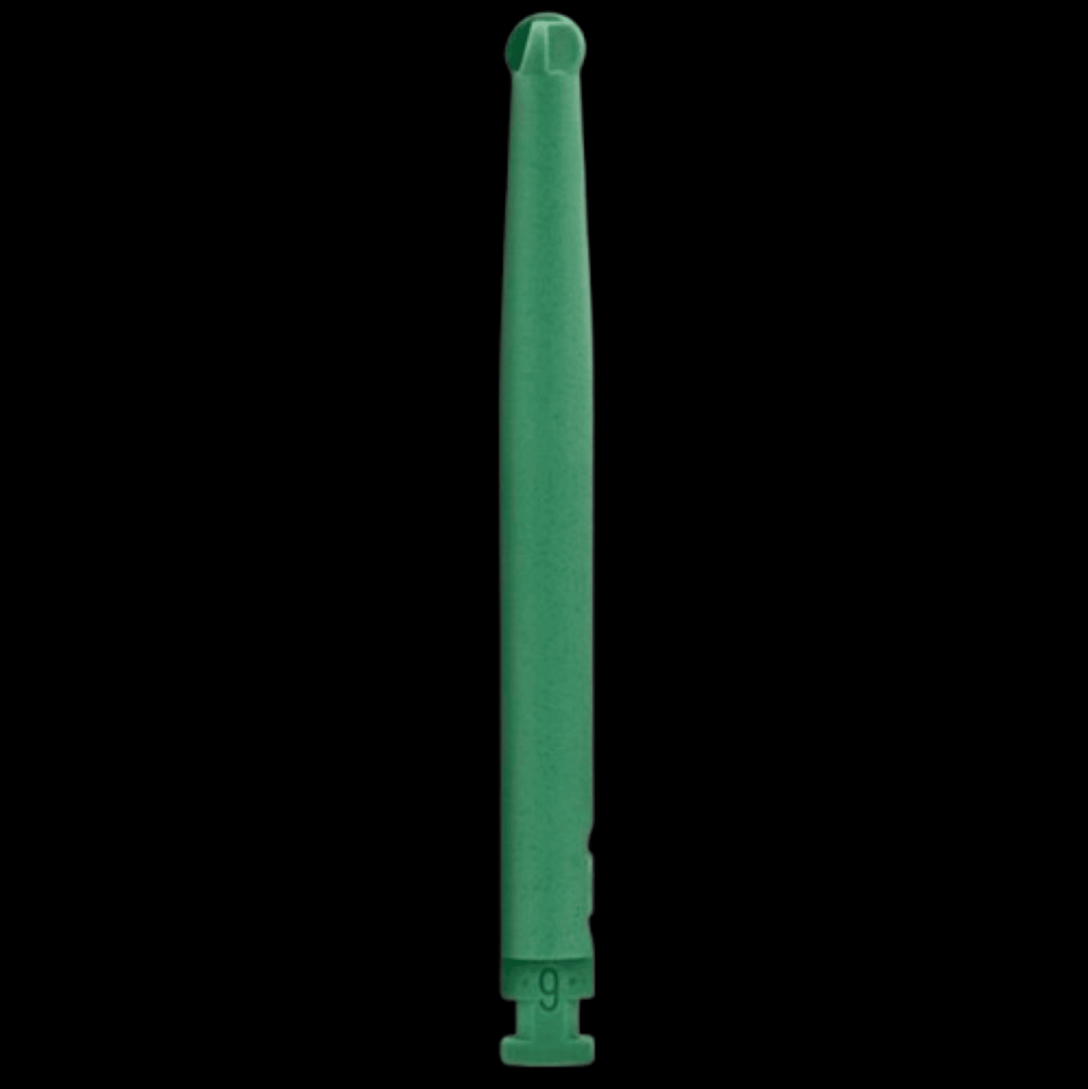
Smart prep bur. The image is captured by, Mausmee Ved, the author of this article. Nickel-titanium (Ni-Ti) rotary instruments

Only defective dentin is removed by these polymer bursts. The dentin that has been injured remains unharmed since it can remineralize. By using these smart preparation burs, infected dentin is removed [23]. Carious dentin is selectively removed using smart burs, protecting good dentin in the process [23]. The polymer cutting edges grow blunt and lose their sharpness when they come into contact with tougher materials, such as healthy dentin [24], as shown in Figure 3.

Nickel-titanium (Ni-Ti) rotary instruments

**Figure 4:**
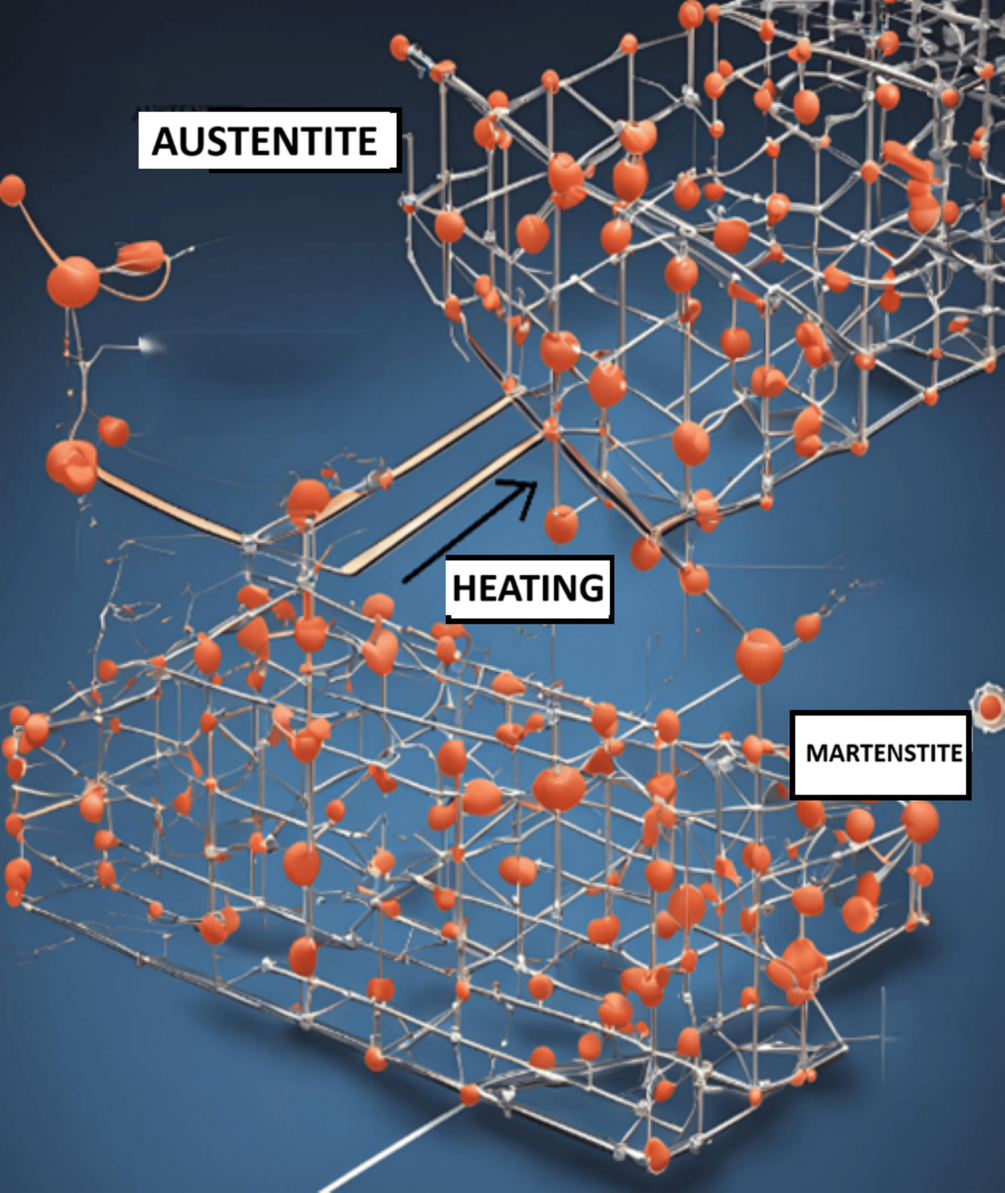
The structure modification is linked to the NiTi alloy's shape memory effect The image is generated by, Mausmee Ved, the author of this article using Canva and Microsoft Paint Microsoft Paint (Redmond, USA), Canva (Sydney, Australia)

Compared to traditional hand instrumentation, the use of Ni-Ti in rotary endodontics has simplified and expedited instrumentation during root canal therapy [25,26]. One of the advantages of rotating Ni-Ti files is that it lowers the risk of canal aberration, operator fatigue, postoperative discomfort, and file breakage during instrumentation [27]. In 1988, Walia et al. brought NiTi to the field of endodontics; for example, NiTi rotary files. During the thermomechanical process, curved root canals can be more easily accessed thanks to the extreme flexibility of NiTi rotary instruments [28]. It permits fewer canal aberrations, less canal transit, and more centered canal preparations. Nitinol changes from an austenitic (strong and hard) crystalline stage to a martensitic (very elastic) arrangement under stress at a constant temperature. Bending just needs a little force during this stage of martensitic formation (Figure 4). The structure reverts to its initial austenitic phase and shape upon the relaxation of the tension [29]. Stress-induced thermoelastic transition is the name given to this phenomenon [30].

Interappointment Intracanal medicaments

Antibacterial and anti-inflammatory drugs that might be utilized between visits are termed intracanal drugs [31]. They are available as pastes, gels, and points that are inserted inside the canal. The most widely used material is calcium hydroxide paste. It causes the creation of hydroxyl ions, which increases the pH in the root canal and harms microbial DNA [32]. When administered alone or in combination with chlorhexidine, calcium hydroxide and silver nanoparticles (size 20 nm) show increased antibacterial activity [33].

Smart antimicrobial peptide

Given their high degree of specificity for microorganisms, which enables them to lessen some of the negative effects of antibiotics, like secondary infections and the eradication of commensal organisms, these compounds may also be categorized as probiotic antibiotics [34,35]. To successfully eliminate the most common strain of *Streptococcus mutans* that causes dental cavities, a pheromone-guided "smart" antimicrobial peptide is being developed [36].

Obturation

Obturation is described as the method used to fill and seal a cleaned root canal in all three dimensions using root canal filling material and sealers [37]. This is accomplished by combining a solid or semisolid bulk filler with a sealer [38]. In the obturation process, the following bulk fillers are frequently utilized: Resilon, gutta-percha (GP), and silver points. GP is an obturating substance that is structurally stable, inert, and biocompatible. Recent formulations have included Bioglass as well as nanoparticles for obtaining GP's proactive features [39]. The mechanical properties (elastic modulus and strength) of the amoxicillin-embedded nano-diamond GP (NDGP) composite were found to be superior to those of the widely utilized gutta percha [39].

Sealers

An efficient three-dimensional in-form seal in the root canal structure requires a mix of endodontic sealers and obturating materials. Even when the root canal is warmed to increase its flow rate, GP is unable to adhere to the root dentin. This defect in GP obturating material necessitates the use of a sealer to create a fluid-tight closure by filling in the spaces between the obturating material and root dentine. Obturating sealers were made with zinc oxide nanoparticles and chitosan [40]. Because outcomes demonstrated that these nanoparticles reduced the entry of bacteria in the canal, it was determined that using them in sealers had a favorable outcome [41]. For use as a sealant, a zinc oxide nanoparticle has been created and is marketed as Nano Seal-S (Prevest DenPro Limited, Jammu, India) [42].

Smart sutures

**Figure 5:**
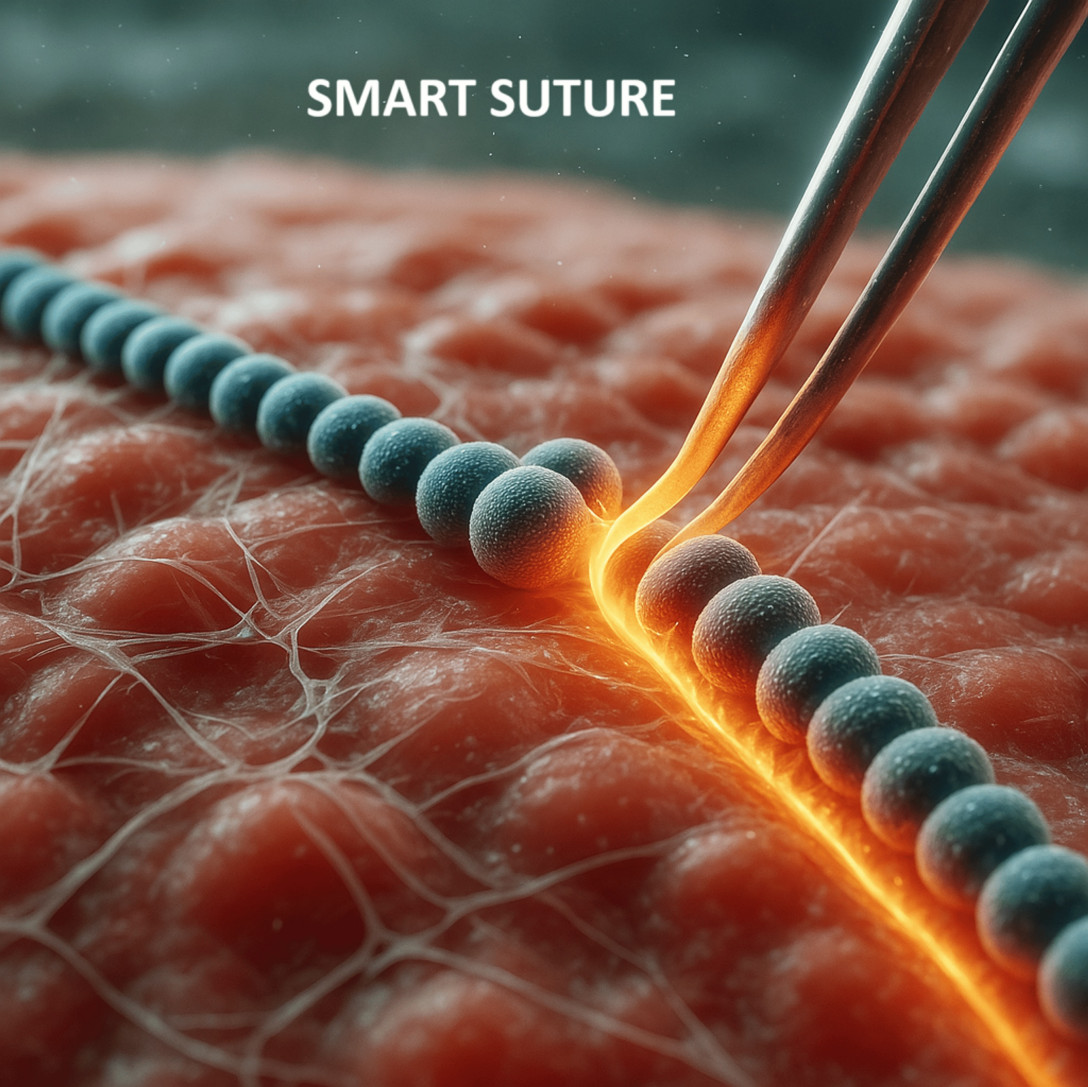
When the temperature increases above the thermodynamic threshold temperature, a smart suture compresses and tightens the knot. The image is generated by, Mausmee Ved, the author of this article using Canva and modified using Microsoft Paint Microsoft Paint (Redmond, USA), Canva (Sydney, Australia)

The thermoplastic polymers used to make these sutures are biodegradable and exhibit form memory [43,44]. The suture ends were secured, and they were applied loosely in their temporary shape. The knot would become firmer as the suture shrank, adding the maximum tension if the temperature were elevated over the thermal transition point (Figure 5). In order to tie a knot with the right amount of force during surgery, this thermal transition temperature is clinically significant because it is comparable to the body temperature of a human [45]. Infections can be detected via silk or as plastic fibers coated with temperature gauges, and micro-heaters make up smart sutures [46].

Smart impression material

They are hydrophilic in order to provide an impression free of space. Their hardness keeps them from tearing, and their shape memory keeps them from distorting during elastic recovery for a more precise impression [47]. They produce accurate, distortion-free fitting restorations because of their snap-set behavior. Hours spent working and setting were cut by at least 33% [48]. Their low viscosity allows them to flow easily. For example, Imprint TM 3 VPS, Impregim TM, and Aquasil Ultra (Dentsply Sirona, Charlotte, NC) [49].

Nanoparticles in dental application

The application of nanoparticles in many dental specialties has grown dramatically since their inception. It is easy to mix these nanoparticles into a sealant to provide the intended effects, irrigating fluids, intracanal medications, and obturating substances. In the scientific field of nanotechnology, materials are manipulated and restructured on a nanoscale scale (less than 100 nanometers) to create new materials with unique features and functions [50].

In 1960, Richard P. Feynman made the initial mention of nanotechnology. The Greek word "nano" means "dwarf." In 1867, Clerk Maxwell created the idea of nanorobots [51]. Unbound natural or synthetic materials or aggregates having particle sizes between one and one hundred nanometers are known as nanomaterials. Among the many properties of nanomaterials are their incredibly reduced diameters, increased chemical reactivity, and a high volume of surface-to-weight ratio. One benefit of nanoparticles is their capacity to engage with human tissue at the chemical and cytoplasmic levels. It was introduced in endodontics to enhance the mechanical integrity, tissue regeneration, and antibacterial activity of dentin that was already compromised [51].

Irrigation "washing by a stream of fluid" is how root canal irrigation is defined, and "intracanal irrigation promotes physical removal of debris from the canal and introduction of chemicals for antibacterial action, demineralization, tissue dissolution, bleaching, and deodorizing and hemorrhage control”.

The three frequently utilized irrigants are sodium hypochlorite (NaOCl), ethylenediaminetetraacetic acid (EDTA), and chlorhexidine (CHX) [52].

Although chlorhexidine is safer than sodium hypochlorite, it does not remove biofilm or smear layers from root canal dentine. Because of the limits of traditional irrigants, nanoparticles have been used to create new irrigation materials [52]. Chitosan nanoparticles have been shown to have improved antibiofilm efficacy and the ability to deactivate bacterial endotoxins. The accelerated bacterial breakdown caused by these nanoparticles can be seen in the organized release of singlet oxygen species [52].

Smart fiber for laser dentistry

In order to produce high-fluence laser light that can ablate dental enamel, orders of picosecond impulses of Nd: YAG laser light focused on a tooth surface are sent via a hollow-core photon crystalline fiber with a core diameter of roughly 14 micrometers [53,54]. The 1.06-micrometer laser's single basic mode phase light is supported by hollow-core photonic crystal fiber (PCF) [55,56]. The same fiber is also used to transmit emissions from plasmas, which are produced by laser beams, for detection and optical diagnostics [57]. The summary of smart dental materials is given in Table 1.

**Table 1:** Summary table: smart materials in restorative dentistry and endodontics The Table is created by the author: Mausmee Ved ACP: Amorphous Calcium Phosphate; Ca: Calcium Ion; PO₄: Phosphate Ion; F⁻: Fluoride Ion; GIC: Glass Ionomer Cement; NP: Nanoparticles; DMADDM: Dimethylaminododecyl Methacrylate; VPS: Vinyl Polysiloxane; Ca(OH)₂: Calcium Hydroxide; AgNP: Silver Nanoparticles; GP: Gutta Percha

Category	Smart Material/Technology	Key Features	Applications
Smart Composites	ACP-based Smart Composites	Releases Ca, PO₄, F⁻ ions; pH-responsive; remineralizes teeth	Class I & II restorations in primary/permanent teeth
Self-Healing Composites	Resin-filled Microcapsule System	Breaks release resin; reacts with catalyst to seal cracks	Extends material life, self-repair ability
Smart Glass Ionomer Cement	Resin-modified GIC	Thermal responsiveness, fluoride recharge	Cavity fillings, liners, bases
Smart Ceramics	CERCON Zirconium	Metal-free, high strength, crack-resistant, aesthetic	Bridges, crowns, implants
Smart Prep Burs	Polymer burs	Selective dentin removal	Caries removal without damaging healthy dentin
Ni-Ti Rotary Instruments	Nickel-titanium alloys	Shape memory, flexibility, reduced operator fatigue	Root canal shaping
Bonding Systems	ACP, Silver NP, DMADDM enhanced adhesives	Antimicrobial, remineralization	Restorative bonding agents
Sealants	Fluoride-releasing sealants	Prevents demineralization, dual-curing	Pit and fissure caries prevention
Sutures	Thermoplastic with shape memory	Temperature-induced tightening, infection detection	Surgical suturing with smart response
Smart Impression Materials	VPS	Hydrophilic, shape memory, snap-set	Dental impressions
Intracanal Medicaments	Ca(OH)₂ + AgNPs	High pH, antimicrobial, DNA damage to bacteria	Between endodontic visits
Smart Antimicrobial Peptides	Pheromone-guided peptides	Targeted action, less disruption to healthy flora	Caries prevention, antimicrobial therapy
Nanoparticles	AgNPs, Chitosan, Bioactive NPs	Antibacterial, enhanced tissue regeneration, drug delivery	Sealers, irrigants, obturating materials
Irrigation	Nano-enhanced solutions (e.g., Chitosan NP)	Biofilm removal, endotoxin deactivation	Root canal cleaning
Smart Fiber	Hollow-core photonic crystal fiber	Laser delivery for ablation, diagnostics	Laser dentistry
Obturation Materials	Nano-diamond GP, Bioglass	Improved mechanical strength, bioactivity	Root canal obturation
Sealers	Zinc oxide NP, chitosan-based	Improved sealing, antibacterial properties	Endodontic sealing

## Conclusions

The creation of these improved and more recent smart materials will fundamentally alter several dental treatment techniques, making them more operator-friendly and patient-comfortable. The intelligence of smart materials is always growing; therefore, it will definitely be a good investment for dentists. A step into the future, A new era of bio-smart dentistry has begun as a result of these developments in material science! In endodontics, nanoparticle-based treatments can improve the bactericidal effect. On the basis of clinical needs, new nanoparticle compositions are being introduced. Nanoparticles should be prioritized in medical research and dentistry for future progress.
